# The Novel Tetra-Specific Drug C-192, Conjugated Using UniStac, Alleviates Non-Alcoholic Steatohepatitis in an MCD Diet-Induced Mouse Model

**DOI:** 10.3390/ph16111601

**Published:** 2023-11-13

**Authors:** Jihye Kim, Nakho Chang, Yunki Kim, Jaehyun Lee, Daeseok Oh, Jaeyoung Choi, Onyou Kim, Sujin Kim, Myongho Choi, Junyeob Lee, Junghwa Lee, Jungyul Kim, Minji Cho, Minsu Kim, Kwanghwan Lee, Dukhyun Hwang, Jason K. Sa, Sungjin Park, Seungjae Baek, Daeseong Im

**Affiliations:** 1 Onegene Biotechnology, Inc., 205 Ace Gwanggyo Tower 2, 91 Changnyong-daero 256 beon-gil, Yeongtong-gu, Suwon-si 16229, Republic of Korea; jihye.kim@onegenebt.com (J.K.); jaeyoung.choi@onegenebt.com (J.C.); jungyul.kim@onegenebt.com (J.K.); kwanghwan.lee@onegenebt.com (K.L.);; 2 Department of Biomedical Sciences, Korea University College of Medicine, Seoul 02841, Republic of Korea; 3 Department of Biomedical Engineering, Ulsan National Institute of Science and Technology, Ulsan 44919, Republic of Korea

**Keywords:** multi-specific, conjugation, platform, complex disease, NASH, GLP-1, GCG, FGF21, IL-1RA, chronic inflammation

## Abstract

Non-alcoholic steatohepatitis (NASH) is a complex disease resulting from chronic liver injury associated with obesity, type 2 diabetes, and inflammation. Recently, the importance of developing multi-target drugs as a strategy to address complex diseases such as NASH has been growing; however, their manufacturing processes remain time- and cost-intensive and inefficient. To overcome these limitations, we developed UniStac, a novel enzyme-mediated conjugation platform for multi-specific drug development. UniStac demonstrated high conjugation yields, optimal thermal stabilities, and robust biological activities. We designed a tetra-specific compound, C-192, targeting glucagon-like peptide 1 (GLP-1), glucagon (GCG), fibroblast growth factor 21 (FGF21), and interleukin-1 receptor antagonist (IL-1RA) simultaneously for the treatment of NASH using UniStac. The biological activity and treatment efficacy of C-192 were confirmed both in vitro and in vivo using a methionine-choline-deficient (MCD) diet-induced mouse model. C-192 exhibited profound therapeutic efficacies compared to conventional drugs, including liraglutide and dulaglutide. C-192 significantly improved alanine transaminase levels, triglyceride accumulation, and the non-alcoholic fatty liver disease activity score. In this study, we demonstrated the feasibility of UniStac in creating multi-specific drugs and confirmed the therapeutic potential of C-192, a drug that integrates multiple mechanisms into a single molecule for the treatment of NASH.

## 1. Introduction

Multi-specific drugs have garnered the attention of the scientific and clinical communities as promising therapeutic strategies owing to their ability to target complex structures, overcoming the limitations of monoclonal antibodies [1]. Complex diseases, characterized by their multifaceted nature, often involve the simultaneous activation of various disease mediators and receptors that are interconnected. Therefore, the development of multi-specific drugs that can target various molecules has emerged as a promising therapeutic approach, as shown by recent advancements in combination therapy and multi-specific antibodies (MsAbs) [2,3]. Combination therapy entails the administration of multiple compounds concurrently to achieve synergistic therapeutic effects. Although such an approach holds great potential for targeting multiple signaling networks at the same time, it is hampered by several limitations, including an increased risk of toxicity and adverse side effects. Furthermore, determining the optimal combination of therapies and appropriate dosing regimens could pose significant challenges [4,5,6]. In contrast, bispecific antibodies (BsAbs) exhibit a unique ability to bind to two different antigens or epitopes simultaneously, enabling the blockade of multiple disease-related pathways. However, the production of BsAbs presents severe challenges due to their complex structural requirements, increased costs, and the potential for heightened immunogenicity, which can trigger adverse immune reactions [7,8,9,10,11,12,13,14].

Non-alcoholic fatty liver disease (NAFLD) accounts for one of the most common liver diseases, and its incidence has been increasing rapidly worldwide [15]. Non-alcoholic steatohepatitis (NASH), a more aggressive form of NAFLD, is strongly linked to obesity, dyslipidemia, and type 2 diabetes. NASH is a complex disease caused by the accumulation of fat in the liver, which subsequently leads to inflammation and cell damage. NASH often progresses to cirrhosis and liver failure, and there is currently no approved medication for its treatment. Therefore, given the multifaceted characteristics of liver diseases, it is essential to develop drugs that can comprehensively target hepatic metabolism and provide multiple benefits, such as improving fatty liver, inflammation, and fibrosis [16,17]. In this study, we aim to propose an innovative approach by simultaneously suppressing several molecular targets that are being therapeutically exploited to improve the treatment of NASH. Glucagon-like peptide-1 (GLP-1) belongs to a class of incretin peptides that enhance glucose-stimulated insulin secretion and reduce food intake [18]. Its analogs are widely being used for the pharmacological therapy of type 2 diabetes and related diseases. Liraglutide, an analog of GLP-1, has been approved by the Food and Drug Administration (FDA) and is currently used as a weight-loss medication [19,20,21,22]. Recently, a treatment strategy using a GLP-1-based multi-target drug has been reported to improve obesity and exhibit higher antidiabetic efficacy compared to GLP-1 receptor agonists alone [23]. Glucagon (GCG) is a peptide hormone secreted by the islet α cells, contributing to the maintenance of euglycemia in humans. Elevated fasting plasma glucose is a biochemical hallmark of diabetes. GCG also increases hepatic glucose production during fasting, which is often associated with NASH progression due to inflammation [24,25]. Moreover, a combination of GLP-1 receptor agonists with GCG exhibits synergistic effects in alleviating weight loss, hepatic inflammation, and fibrosis [26,27,28,29,30,31]. Fibroblast growth factor 21 (FGF21) is secreted primarily in the liver and regulates glucose levels and lipid metabolism. FGF21 has been speculated to be highly effective in alleviating insulin resistance and improving the function and quality of metabolism damage-related cells. In previous animal studies, FGF21 was found to be essential for the weight control effect of glucagon agonists and attenuated hepatic steatosis, inflammation, and fibrosis in a NASH mouse model [32,33,34,35,36,37,38]. Additionally, a triple agonist targeting GLP-1, glucose-dependent insulinotropic polypeptide (GIP), and GCG receptor (GCGR) promoted considerable weight loss, accompanied by increased plasma levels of FGF21 due to GCGR signaling [39,40,41]. The interleukin (IL)-1 family of cytokines is one of the main drivers of inflammation in NASH [42,43,44]. Upon activation, these proinflammatory cytokines can disrupt insulin sensitivity and lipid signaling pathways. Among them, IL-1β plays a crucial role in liver diseases, being involved in all essential stages, from liver inflammation to liver fibrosis [45]. Interleukin-1 receptor antagonist (IL-1RA) is a naturally occurring protein that preferentially binds to the IL-1 receptor. Moreover, IL-1β is the main target of IL-1RA given its functional role in inducing the NLR family protein containing a pyrin domain 3 (NLRP3) inflammasome pathway, contributing to chronic inflammation and fibrotic response in the liver [46,47,48,49]. Therefore, we speculated that IL-1RA could be further employed as a therapeutic target for the development of a multi-target drug eliciting synergistic effects in inhibiting liver inflammation.

In the present study, to overcome the limitations of the traditional multi-target platform, we developed UniStac, a conjugation system based on the ubiquitination mechanism involving three enzymes, namely, ubiquitin-like modifier activating enzyme 1 (UBA1), ubiquitin-conjugating enzyme E2 N (UBC13), and ubiquitin-conjugating enzyme variant MMS2 (MMS2) [50,51]. UniStac produces isopeptide bonds that generate a Y-shape branched molecule [52,53,54]. Moreover, compared to BsAb or MsAb platforms, UniStac provides several advantages, primarily the elimination of homodimer formation and the Fc-heterodimerization optimization steps. And we used the UniStac platform to generate a multi-specific drug candidate, C-192, that binds to GLP-1, GCG, FGF21, and IL-1RA simultaneously. We assessed the pharmacological activity of C-192 using in vitro cell-based assays. Furthermore, the anti-steatosis and anti-inflammatory effects of C-192 were investigated in methionine-choline-deficient (MCD) diet mouse models with profound efficacies.

## 2. Results

### 2.1. Conjugation of Multiple Targets Using a Novel Enzyme-Mediated Platform—UniStac

**Figure 1:**
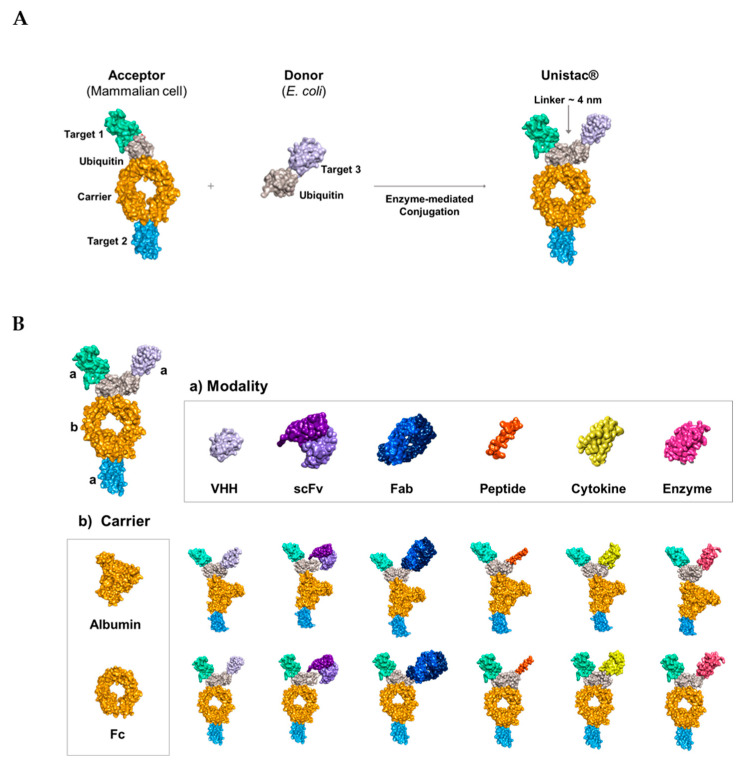
Schematic representation of the overall strategy for the development of UniStac-based multi-specific molecules. (**A**) The UniStac platform is composed of an acceptor molecule (which produces mammalian cells) and a donor molecule (expressed in *Escherichia coli*). The acceptor and donor molecules are conjugated by enzymes and form a Y-shaped antibody-like protein. (**B**) The a is modality and the b is carrier of drug. The UniStac platform can conjugate any modalities (VHH, scFv, Fab, peptide, cytokine, and enzyme) and carriers (albumin or Fc region).

We established an enzyme-mediated platform approach for the generation of multi-specific target molecules. The UniStac platform employs the ubiquitin conjugation system to generate Y-shaped antibody-like molecules. The main feature of the UniStac platform is its ability to independently produce two proteins (acceptor and donor) and combine them through an enzyme-mediated reaction to yield a tri- or tetra-specific target drug. The UniStac platform uses acceptor molecules with acceptor ubiquitin (C-terminus blocked for activation and only one surface lysine [Lys] available for conjugation) and donor molecules with donor ubiquitin (C-terminus available for ubiquitin activation and ablation of all Lys residues) [55,56]. In contrast to previous enzyme classes such as sortases (head-to-tail), the ubiquitin enzymes (UBA1, UBC13, and MMS2) form an isopeptide bond (branched), and the enzyme-mediated reaction results in a Y-shaped antibody-like protein (Figure 1A) [57,58]. Under mild conditions (pH 7.0, 25 °C), UniStac-mediated protein conjugation achieves >95% yield, regardless of the modalities. The platform accommodates various carriers, including albumin or the Fc region, and diverse target modalities such as the variable domain of heavy-chain antibodies (VHH), enzymes, and single-chain variable fragments (scFvs) with equal conjugation efficiency (Figure 1B). This enables streamlined multi-target drug development. Therefore, by altering the acceptor and donor targets, a new triple-targeted compound can be developed, generating multi-specific molecules with flexible valency via a straightforward process (Figure S1).

### 2.2. Development of the Novel Tetra-Specific Drug C-192

**Figure 2:**
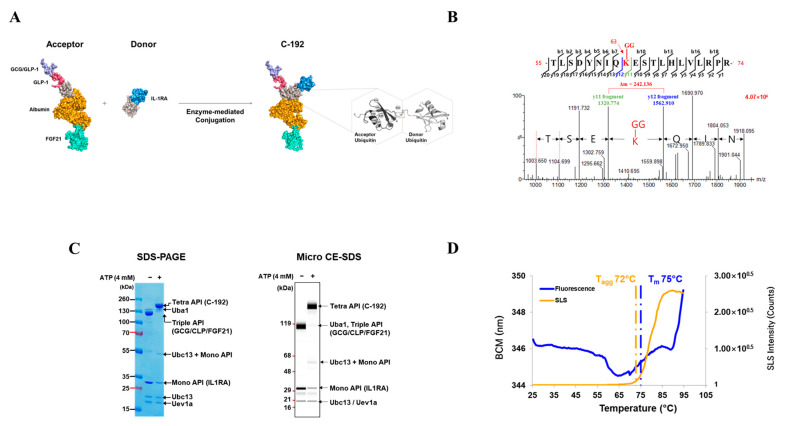
Characterization of C-192 developed using the UniStac platform. (**A**) C-192 was synthesized using the UniStac platform with site-specific conjugation of lysine and glycine. (**B**) The spectrum of the C-192 conjugation site was determined using liquid chromatography with tandem mass spectrometry (LC-MS/MS). The ubiquitination site was extremely specific, i.e., between Lys63 of the acceptor and the C-terminal of the donor ubiquitin. (**C**) The high conjugation yield of C-192 was confirmed using sodium dodecyl sulfate-polyacrylamide gel electrophoresis (SDS-PAGE) and microcapillary electrophoresis-sodium dodecyl sulfate (CE-SDS). (**D**) The stability of C-192 was determined using differential scanning fluorimetry (DSF). The unfolding transition temperature (T_m_) was detected using fluorescence (blue), and the aggregation temperature (T_agg_) was detected using static light scattering (SLS, yellow).

We improved the physical properties and cleavage resistance of the FGF21, GLP1, and GCG targets by engineering their sequences [27,34,59]. We fused GLP1/GCG and FGF21 to the N- and C-termini of ubiquitin-albumin, respectively, to generate an acceptor molecule expressed in mammalian cells. For the donor molecule, *Escherichia coli* cells were used to produce IL-1RA, and its amino acid sequence was fused to the N-terminus of ubiquitin. The acceptor and donor proteins were conjugated using the enzyme-mediated UBA1/UBC13/MMS2 conjugation system, UniStac, via Lys63-linked diubiquitin to form a C-192 molecule (Figure 2A) [51]. The acceptor, donor, UBA1, UBC13, and MMS2 were combined in a 1:1.3:0.1:0.5:0.5 ratio, and ATP was added to initiate the conjugation reaction. To confirm site-specific ubiquitination within the C-192 molecule, we performed liquid chromatography with tandem mass spectrometry (LC-MS/MS) assay, which revealed that GlyGly (114.04 Da) modification occurred only on a single Lys residue of the acceptor ubiquitin. As shown in Figure 2B, the ubiquitination occurred on the Lys63 residue of the peptide (55-TLSDYNIQKESTLHLVLRPR-74), carrying the engineered acceptor ubiquitin. The Y11 fragment, with a mass of 1320.77 Da, corresponds to the sequence ESTLHLVLRPR-74 following the modified Lys residue, and the Y12 fragment, with a mass of 1562.91 Da, corresponds to the sequence K(-GG)ESTLHLVLRPR-74, which includes the ubiquitinated Lys residue. The difference of 242.136 Da in mass between Lys (146.2 Da) and GlyGly modification (114.04 Da), excluding the H_2_O mass, was confirmed as the mass difference between Y11 and Y12 fragments, indicating ubiquitination of the Lys63 residue.

The conjugation yield between the acceptor and donor molecules was measured at >95% through sodium dodecyl sulfate-polyacrylamide gel electrophoresis (SDS-PAGE) and microcapillary electrophoresis-sodium dodecyl sulfate (CE-SDS). We confirmed the absence of both acceptor and donor bands, and the resulting C-192 band was observed at the expected molecular weight of 132.4 kDa, whereas the enzyme bands remained relatively consistent. Micro-CE-SDS analysis revealed that UniStac conjugation was highly efficient, with a >95% yield of C-192 (Figure 2C). Next, we performed differential scanning fluorimetry (DSF) to confirm the thermal stability of C-192. The midpoint temperature of the thermal unfolding transition (T_m_) was measured at 75 °C. Additionally, the aggregation onset temperature (T_agg_) of C-192 at the given formulation was 72 °C (Figure 2D).

### 2.3. In Vitro Potencies of C-192 on Target Molecules

**Figure 3:**
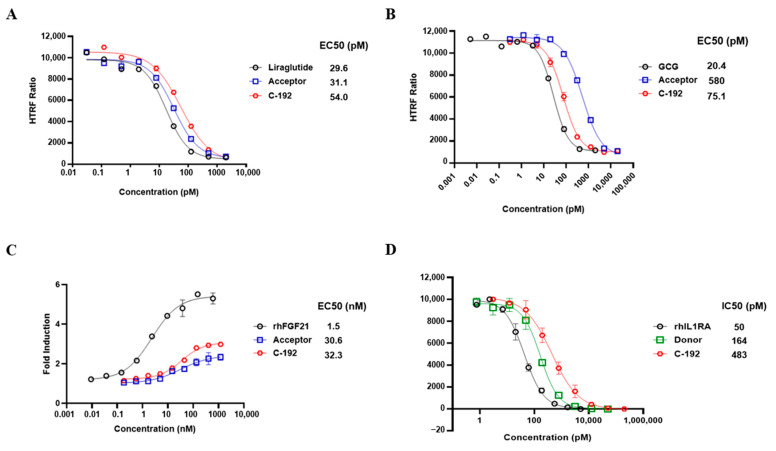
Sustained biological activity of each active pharmaceutical ingredient (API) of C-192. Activities of (**A**) glucagon-like peptide-1 (GLP-1), (**B**) glucagon (GCG), (**C**) fibroblast growth factor 21 (FGF21), and (**D**) interleukin-1 receptor antagonist (IL-1RA). The acceptor is a form in which GLP-1, GCG, and FGF21 are conjugated, and IL-1RA is the donor. GLP-1 and GCG activities were measured using the cyclic adenosine monophosphate (cAMP) accumulation assay in stable CHO-K1 cell lines. FGF21 activity was determined using the FGFR1/KLB functional assay in the U2OS cell line. IL-1RA activity was measured using the nuclear factor kappa B (NF-κB) inhibition assay in the HepG2 cell line.

Surface plasmon resonance (SPR) analysis was used to confirm the lack of interference among target molecules when introduced to the UniStac platform (Table 1). We further investigated whether the vertex of the Y-shaped structure interferes with one another, suggesting the preservation of biological activity upon sequential addition of each molecular target to the UniStac platform. The activities of GLP-1 and GCG were evaluated using the cAMP accumulation assay. We confirmed that both GLP-1 (EC_50_ = 54 pM) and GCG (EC_50_ = 75.1 pM) retained their molecular activities when combined within C-192 (Figure 3A,B). The EC_50_ of FGF21 was 32.3 nM as per the FGFR1/βKlotho (KLB) functional assay. Next, we investigated the inhibitory effects of IL-1RA on the nuclear factor kappa B (NF-κB)-mediated signaling activity through an NF-κB reporter assay. The experimental results revealed an IC_50_ value of 483 pM. Moreover, we evaluated the pharmacological effect of UniStac conjugation on the biological activity of mono-, double-, and triple-conjugated molecules and observed that conjugation did not attenuate their activities, as demonstrated by the consistent biological activities observed in all conjugated forms.

### 2.4. Downstream Regulatory Mechanism of Tetra-Specific Targets in C-192

**Figure 4:**
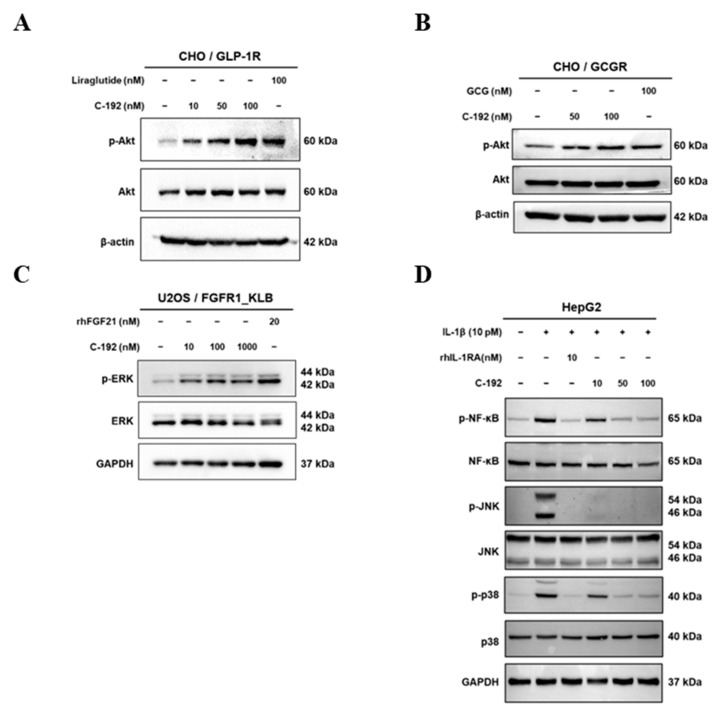
Regulation of downstream signaling by each target. The downstream cellular signaling of four distinct mechanisms by C-192 was examined using western blot analysis. (**A**) GLP-1, (**B**) GCG, (**C**) FGF21, and (**D**) NF-κB and JNK pathways.

To examine the inhibitory effect of C-192 on each target-related molecule, we performed immunoblot analyses of essential proteins involved in corresponding pathways. C-192 treatment resulted in a dose-dependent increase in the phosphorylation activity of serine/threonine kinase (AKT), a downstream protein of both GLP-1 and GCG (Figure 4A,B). C-192 also promoted hyperphosphorylation of ERK, a key downstream molecule of FGF21 (Figure 4C). In the case of IL-1RA, the intracellular inflammatory response is linked to NF-κB activation through the mitogen-activated protein kinase (MAPK) pathway. Major encoding proteins of the MAPK signaling pathway consist of jun N-terminal kinase (JNK) and p38 kinase, which are activated by lipopolysaccharide (LPS) or cytokines and subsequently lead to NF-κB activation [44,45]. In the recombinant IL-1β-induced inflammatory response of the HepG2 cells, the phosphorylation activities of p38, JNK, and NF-κB were coherently suppressed in a dose-dependent manner (Figure 4D). These results collectively suggest that the UniStac conjugation process does not interfere with the molecular activities of its target proteins and provides innovative therapeutic applications in regulating target signaling pathways.

### 2.5. Pharmacokinetic Profile of C-192 and its Therapeutic Effects on the MCD Diet-Induced NASH Mouse Model

**Figure 5:**
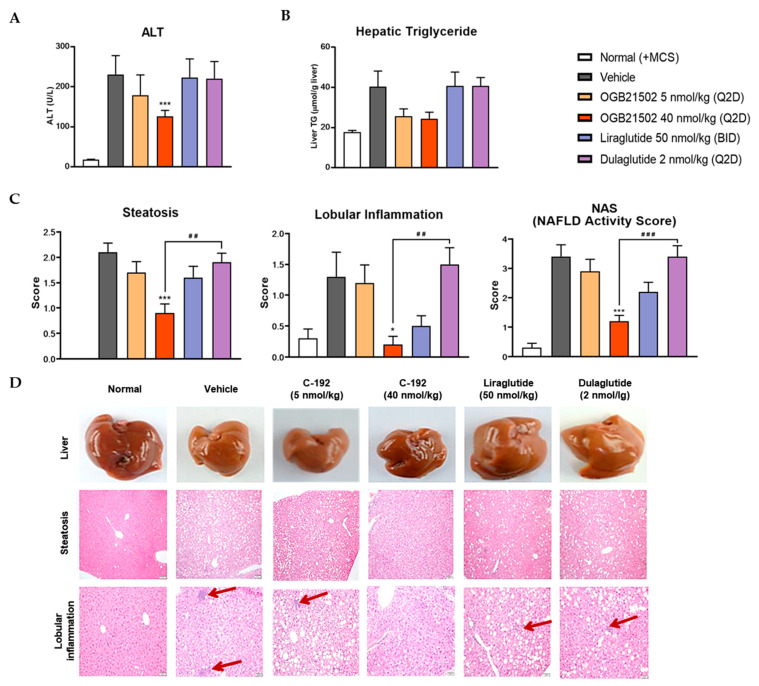
Effect of C-192 on hepatic steatosis and liver injury in a methionine-choline-deficient (MCD) diet-induced non-alcoholic steatohepatitis (NASH) mouse model. Liraglutide and dulaglutide were used as comparative controls. The samples were harvested from mice 4 weeks after administration of C-192. Analysis of (**A**) plasma alanine transaminase (ALT) levels, (**B**) hepatic triglyceride (TG) levels, and (**C**) steatosis-lobular inflammation and NAFLD activity score (NAS). (**A**–**C**), the values are the means ± SEMs (*n* = 10). (**D**) Representative mouse liver tissues and histological photomicrographs of hematoxylin- and eosin-stained liver sections. The red arrows indicate lobular inflammation. * *p* < 0.05, *** *p* < 0.001 compared with vehicle control, and ## *p* < 0.01 and ### *p* < 0.001 compared with dulaglutide.

To interrogate the pharmacokinetic profile of C-192, we generated human albumin serum (HAS)-binding UniStac and performed single-dose experiments in BALB/c mice and cynomolgus monkeys. Non-compartmental analysis (Table 2) revealed that C-192 was suitable for preclinical testing in various animal models and can be provided as a multifunctional agonist for therapeutic purposes that can be administered once a week. To confirm the therapeutic efficacy of C-192 in the treatment of NASH, we established MCD diet-induced mouse models. MCD diet-fed mice demonstrated significantly higher levels of serum alanine transaminase (ALT) and lipid accumulation compared to the control mice, indicative of hepatic steatosis and inflammation. Notably, the C-192 treatment resulted in a dose-dependent reduction in serum ALT and triglyceride levels, compared with the reference drugs liraglutide and dulaglutide (Figure 5A,B). Additionally, histopathological hepatic sections stained with hematoxylin and eosin revealed marked improvements in liver steatosis, lobular inflammation, and NAFLD activity scores (NAS) (Figure 5C,D). These results underscore C-192 as a potential therapeutic candidate for hepatic steatosis and inflammation in an MCD diet-induced NASH.

## 3. Discussion

The development of multi-specific drugs targeting various cytokines and hormones presents several challenges. They consist of complex structures associated with inefficient production processes as well as biophysical interferences stemming from their large molecular sizes [10,60,61]. However, multi-specific drugs provide enhanced therapeutic efficacy against multi-target diseases, underscoring the need for innovative solutions to overcome the existing challenges [62]. Therefore, in the present study, we developed UniStac, a novel enzyme-mediated protein–protein conjugation platform to facilitate the generation of multi-specific molecules [50,51,63,64]. The UniStac platform offers several advantages, including a highly site-specific conjugation process that minimizes undesired homodimer formation, ensuring the preservation of the intended molecular structures. The manufacturing process using UniStac has proven to be efficient in terms of development time, cost, and high conjugation yield. More importantly, the conjugation of ligands did not interfere with their original biological functions, further distinguishing the clinical utility of the UniStac platform for multi-specific drug development.

As its first product, we designed C-192, which simultaneously targets GLP-1, GCG, FGF21, and IL-1RA for the treatment of NASH. GLP-1 is commonly used to treat type 2 diabetes and related diseases [20]. We speculate that combining GLP-1 with GCG, FGF21, and IL-1RA can further enhance the therapeutic efficacy of its treatment. While GCG helps with both weight loss and inflammation [25,29], FGF21 improves metabolism activity and liver health [33,35]. Moreover, IL-1RA inhibits liver inflammation by blocking IL-1β and inflammasome pathways, which have been speculated to be the major drivers of various liver diseases [45]. Among the various pathological mechanisms involved in NASH development, we focused on chronic inflammation and the NLRP3-inflammasome, as they constitute a central role in fibrogenesis [49]. A previous study highlighted that IL-1RA could be more effective in treating patients with severe fibrotic NASH compared to the current standard treatment [26]. The functional evaluation of the UniStac conjugation platform on the biological activity of C-192 revealed unprecedented insights for the development of multi-target drugs. Both GLP-1 and GCG activate the AKT pathway, which results in various cellular responses, including enhanced glucose uptake, insulin secretion, and inhibition of apoptosis. Additionally, FGF21 activates ERK, leading to diverse cellular responses such as cell proliferation, differentiation, and survival. We investigated the therapeutic effect of C-192 on the phosphorylation activities of p38, JNK, and NF-κB in response to a recombinant IL-1β-induced inflammatory response in HepG2 cells and observed a dose-dependent reduction. The preservation of biological activities during the conjugation process is the apogee of this platform, offering significant advantages over traditional drug development and the possibility of improved therapeutic outcomes for complex diseases. C-192, despite its complex structure and a limited number of animal subjects, demonstrated stable physical properties and acceptable pharmacokinetic profiles [65,66,67,68], highlighting the clinical feasibility of UniStac. C-192 presents an ideal PK profile, making it suitable for weekly administration in clinical practice. The biological activity of each molecular target on C-192 was efficiently preserved after conjugation. This may be attributable to the sufficient space within the diubiquitin linker—a useful feature of the UniStac platform.

In the MCD diet-induced steatohepatitis mouse model, C-192 was highly effective in treating hepatic steatosis and inflammation-associated NASH, as all of the mice exhibited complete recovery. Furthermore, C-192 demonstrated superior treatment effects compared to reference GLP-1R agonists, including liraglutide and dulaglutide. Further studies are warranted to elucidate the synergistic impact and mechanistic process of GLP-1/GCG, FGF21, and IL-1RA in C-192. Moreover, its therapeutic potential against fibrosis must be further investigated in fibrotic mouse models.

Collectively, our study highlights the feasibility of UniStac as an optimal platform for the development of multi-target drugs. UniStac offers both time- and cost-effective methods for drug development owing to (1) a highly site-specific conjugation process that minimizes homodimer formation, which ensures that the conjugated molecules maintain their intended structures; (2) an efficient manufacturing process with high conjugation yields; and (3) a lack of interference between target ligands after conjugation, suggesting that the platform is capable of efficiently combining two distinct proteins for multi-targeting purposes without compromising their individual activities. C-192, the resulting molecule from the UniStac platform, demonstrated remarkable potential for the treatment of NASH, and further studies exploring the functional role of C-192 in treating complex diseases, such as liver fibrosis, could reveal additional therapeutic applications of this novel multi-target drug. Although further investigations are warranted to fully elucidate the synergistic effect of IL-1RA on liver fibrosis and the NLRP3-inflammasome mechanism, the results of this study establish the therapeutic potential of C-192 for the treatment of fibrosis. Overall, the UniStac platform represents a promising and innovative approach to the development of multi-target drugs.

## 4. Materials and Methods

### 4.1. LC-MS/MS and Protein Purification

Peptides of C-192 were separated using an ultra-performance liquid chromatography (UPLC) ACQUITY PREMIER system (Waters, Milford, MA, USA). Mobile phase A contained 0.1% formic acid and 99.9% aqueous solution; mobile phase B contained 0.1% formic acid and 99.9% acetonitrile solution. The chromatographic column was equilibrated using 100% solution A. The digested peptide sample was loaded and separated using an ACQUITY UPLC BEH C18 column (1.7 μm, 2.1 × 150 mm; Waters) at a flow rate of 300 nL/min. The UPLC gradients were as follows: solution B, a linear gradient from 5 to 40% for 1–75 min, solution B maintained at 90% for 75–85 min, and equilibration using 5% solution B for 85–90 min. Ionized peptides were analyzed using a Synapt XS mass spectrometer (Waters). MS detection was performed in the positive ion mode. The scan range of the precursor ion was 50–2000 *m*/*z* in the MSE continuum mode.

UniStac molecules were characterized using SDS-PAGE. Automated capillary electrophoresis (CE) was performed using an Agilent 2100 Bioanalyzer (Agilent Technologies, Santa Clara, CA, USA). For CE analysis, a protein analytic solution mixture was loaded onto a microfluidic protein chip and separated by molecular weight using a Bioanalyzer Protein 230 assay kit (Agilent Technologies, #5067-1518) under reducing and non-reducing conditions, according to the manufacturer’s protocol.

### 4.2. DSF Analysis

Melting and aggregation temperatures for C-192 were measured using an Uncle system (Unchained Labs, Pleasanton, CA, USA). The T_m_ was obtained by measuring the barycentric mean fluorescence, and the Tagg was determined from static light scattering measurements at a wavelength of 266 nm during a temperature ramp from 25 °C to 95 °C with 0.6 °C increments. The equilibration time was set to 60 s before each measurement. The concentration of both samples was 1.0 mg/mL. Measurements were made in 10.0 mM histidine (pH 7.0) containing 230.0 mM trehalose (T-104-4, Pfanstiehl Inc., Waukegan, IL, USA). Measurements were made in duplicates and then averaged, and standard errors were calculated using Uncle analysis software v4.01.

### 4.3. Affinity Measurement

SPR was performed using a Biacore™ T200 (GE Healthcare, Stockholm, Sweden) system to measure the binding affinities of GLP-1, GCG, and KLB. The ligand (C-192 or IL-1R1) was diluted in a 10-mM sodium acetate solution (pH 5.5) with a final concentration of 5 or 7.59 μg/mL, respectively. C-192 or IL-1R1 was immobilized on a CM5 sensor chip (GE Healthcare, #50-105-5274) using amine coupling to reach target densities of 400 or 700 resonance units, respectively. The analytes present in C-192 were GLP-1R, GCG, and KLB. The commonly administered concentrations of GLP-1R and GCGR were 3.906, 7.813, 15.626, 31.25, 62.5, 125, 250, 500, and 1000 nM at a flow rate of 50 μL/min for 4 min. KLB (0.195, 0.391, 0.781, 1.563, 3.125, 6.25, 12.5, 25, 50, 100, and 200 nM) and IL-1RA (1.953, 3.903, 7.813, 15.625, 31.25, 62.5, 125, 250, and 500 nM) were injected at a flow rate of 50 μL/min for 4 min. The dissociation time was 10 min for GLP-1, GCG, and KLB and 60 min for IL-1R1. To calculate the equilibrium dissociation rate constants (ka, kd, KD), we used a 1:1 kinetic binding model (A + B ⇌ AB).

### 4.4. In Vitro Biological Assays

GLP-1R- and GCGR-stable CHO-K1 cell lines were acquired from GenScript (Piscataway, NJ, USA) and maintained according to the vendor’s protocols. Cellular cAMP was quantified using the cAMP Gs Dynamic Kit (Cisbio, #62AM4PEB, Bedford, MA, USA). The cell lines were seeded in 96-well half-area plates at a density of 10,000 cells/well and incubated in a 5% CO_2_, 37 °C humidified incubator for 24 h. The cells were treated with serially diluted references or samples for 30 min at 37 °C in 5% CO_2_. After adding the homogeneous time-resolved fluorescence (HTRF) reagents, the plates were incubated for 1 h in the dark, followed by measurement of the HTRF ratio (665/620 nm × 10^4^). LX-2 and HepG2 cells were maintained in Dulbecco’s modified Eagle’s medium (DMEM) supplemented with 10% fetal bovine serum (FBS) and antibiotics (100 U/mL penicillin, 100 µL/mL streptomycin, and 0.25 µL/mL amphotericin B). All cells were cultured at 37 °C in 5% CO_2_. For the FGF21 functional assay, U2OS cells co-expressing human FGFR1 and KLB (Discover X, Fremont, CA, USA) were cultured in AssayComplete™ Cell Culture Kit-103 (Discover X, pro#92-3103G) supplemented with 0.25 µg/mL puromycin, 250 µg/mL hygromycin B, and 500 µg/mL of G418. Subsequently, the cells were plated in 96-well half-area plates at a density of 20,000 cells/well, and serially diluted recombinant human FGF21 and test samples were immediately added to the cells (50 µL final volume per well; performed in triplicate). After 24 h of incubation at 37 °C, PathHunter Detection Reagent (Discover X, #93-0001) was added to the wells, followed by incubation for 1 h at room temperature. For IL-1RA activation, the NF-κB reporter (Luc)-HEK293 cells (BPS Bioscience, San Diego, CA, USA) were cultured in a minimum essential medium supplemented with 1% antibiotic/antimycotic, 10% heat-inactivated FBS, and 50 µg/mL of hygromycin B. The cells were plated in 96-well half-area plates at a density of 20,000 cells/well 24 h prior to the experiment. Recombinant human IL-1RA and test samples were serially diluted and added to the cells (50 µL final volume per well; triplicate) with 5 pM of recombinant human IL-1β. After 24 h of incubation at 37 °C, the expression of the NF-κB gene was analyzed using the One-Glo luciferase assay system (#E6110, Promega, Madison, WI, USA).

### 4.5. Western Blotting Analysis

Cells (CHO, U2OS, and HepG2) were lysed in RIPA lysis buffer (Sigma Aldrich, Hamburg, Germany) mixed with a 1X protease inhibitor cocktail (Roche, Basel, Switzerland). Proteins were resolved using SDS-PAGE and transferred to polyvinylidene difluoride membranes. The membranes were blocked using 5% skim milk (Becton Dickinson, NJ, USA) and then incubated overnight at 4 °C with 1:1000 diluted phospho-specific and total antibodies against the following proteins: JNK (#9252S), p38 (#8690S), pNF-κB P65 (#3033S), AKT (#9272S), phospho-JNK (Thr183/Tyr185) (#9255S), and phospho-p38 (Thr180/Tyr182) (#4511S) (Cell Signaling Technology Inc., Beverly, MA, USA). NF-κB (#PA1-86), Phospho-AKT (Ser473) (#700392), ERK (#MA5-15134), phospho-ERK (Thr202/Tyr204) (#14-9109-82) antibody were acquired from Invitrogen (Waltham, MA, USA). Recombinant proteins of an agonist or antagonist, rhIL-1RA (#280-RA-101), rhIL-1β (#201-LB) and rhFGF21 (#2539-FG), were acquired from R&D Systems (Minneapolis, MN, USA). Liraglutide a GLP-1 receptor agonist (#6517), and GCG, a ligand for the GCG receptor (#6927), were acquired from TOCRIS (Bristol, UK). The protein bands were visualized using Clarity™ Western ECL Substrate (#1705060, Biorad, Hercules, CA, USA) after hybridization with 1:5000 diluted HRP-conjugated secondary antibodies from rabbits (Invitrogen, #31460) or mice (#GTX213111-01, Genetex, Irvine, CA, USA) and detected using the iBright (Thermo Fisher, Waltham, MA, USA) imaging system.

### 4.6. Pharmacokinetics of C-192 in Animal Models

The pharmacokinetic parameters following a single subcutaneous administration of C-192 were evaluated in male C57BL/6 mice (NDIC Co., Ltd., Hwaseong, Republic of Korea) and cynomolgus monkeys (GENIA Co., Ltd., Gyeonggi-do, Republic of Korea). Animal experiments were performed following the animal welfare guidelines of the Laboratory Animal Care Procedures of NDIC and GENIA. The pharmacokinetic parameters of C-192 were evaluated in C57BL/6 mice (*n* = 3) and cynomolgus monkeys (*n* = 1) at doses of 10 and 15 mg/kg, respectively. Blood samples (600 to 1000 μL) were collected from the animals at the following time points: 2, 4, 8, 12, 16, 24, 36, 48, 72, 96, and 120 h after administration. Additionally, blood samples from the monkeys were collected and analyzed at 144, 168, 192, and 216 h after administration. Plasma samples were collected before and after dosing at the indicated times and stored at −80 °C until enzyme-linked immunosorbent assays and electrochemiluminescence immunoassays.

### 4.7. MCD Diet-Induced Mouse Model

Animal experiments were conducted at Biotoxtech Co. and were approved by the Institutional Animal Care and Use Committee (IACUC) of Biotoxtech Co. (Approval no: 190520) using male C57BL/6 mice (7-week-old; Biotoxtech Co., Ltd., Chungju-si, Republic of Korea). The mice were housed in a pathogen-free facility under controlled conditions. They were randomly divided into six experimental groups: methionine-choline-sufficient (MCS) diet-fed, MCD diet-fed, and four drug-treated groups (*n* = 10). The drugs used were C-192 (5 and 40 nmol/kg), liraglutide (50 nmol/kg), and dulaglutide (2 nmol/kg). All mice were fed either the MCS or MCD diet for 8 weeks, after which drug administration was initiated. The treatment was continued for an additional 4 weeks.

### 4.8. Measurement of ALT Levels

Terminal blood samples were collected in heparinized tubes. Samples were then centrifuged for 5 min at 3000 rpm at 4 °C to separate and stored at −80 °C until analysis. Plasma ALT levels were measured using a Hitachi 7180 analyzer (JSCC, Tokyo, Japan) according to the manufacturer’s instructions.

### 4.9. Histological Analysis

Mouse liver samples were fixed in 10% (*w*/*v*) phosphate-buffered formalin for 48 h. After dehydration using a graded series of ethanol solutions, the liver tissue samples were embedded in paraffin wax. Tissue sections were cut by a microtome, and terminal samples were stained using hematoxylin and eosin to investigate hepatic morphology, including steatosis and inflammation. NAS was calculated by adding the scores of steatosis, lobular inflammation, and hepatocellular ballooning. Blinded liver histology was scored using the NAS criteria [69]. Hepatic TG levels in terminal liver samples were determined using a Triglyceride Reagent Kit (#ETGA200, BioAssay Systems, Hayward, CA, USA) according to the manufacturer’s protocol.

### 4.10. Statistical Analysis

Statistical analyses were performed using PRISM v8.0 (GraphPad Software, San Diego, CA, USA). A one-way analysis of variance was used to evaluate statistical significance. Multiple comparisons were performed between the vehicle and positive control groups to determine significant differences between the control and test groups. Statistical significance was set at *p* < 0.05.

## 5. Conclusions

Taken together, we have developed C-192, a novel tetra-specific drug, through UniStac conjugation technology for the treatment of NASH. UniStac, an enzyme-mediated conjugation platform, has exhibited remarkable conjugation efficiency, thermal stability, and the preservation of biological activity, establishing its potential as a valuable tool for multi-target drug development. C-192 comprises four targets: GLP-1, GCG, FGF21, and IL-1RA. Importantly, our research confirms that the biological activity of each target is sustained without interference during the conjugation process. Moreover, in the MCD diet-induced mouse model, C-192 exhibited remarkable efficacy by significantly reducing ALT levels and hepatic triglyceride accumulation, effectively alleviating steatosis and inflammation. Based on this study, the importance of these results suggests their usefulness in discovering new multi-target drugs to treat complex diseases in the future. We demonstrate the potential of C-192 as an important alternative for treating severe NASH with lipid accumulation and inflammation in the liver due to the synergistic effects across multiple targets.

## Figures and Tables

**Table 1:** Kinetic results of samples binding to GLP-1, GCG-1, IL-1RA, and FGF21 with a 1:1 binding model.

Ligand	Analyte	K_a_ (1/Ms)	K_d_ (1s)	K_D_ (M)	Rmax (RU)	Chi^2^ (RU^2^)
C-192	GLP-1R	1.122 × 10^4^	9.537 × 10^−3^	8.500 × 10^−7^ (850 nM)	34.62	1.13
	GCGR	2.630 × 10^4^	5.108 × 10^−4^	1.942 × 10^−8^ (19.42 nM)	9.699	0.141
	β-Klotho	2.279 × 10^5^	3.831 × 10^−3^	1.681 × 10^−8^ (16.81 nM)	175.7	69.6
	IL-1R1	2.017 × 10^4^	6.779 × 10^−5^	3.360 × 10^−9^ (3.36 nM)	77.64	1.85

**Table 2:** Preliminary pharmacokinetic parameters of C-192 subcutaneous administration in mice and monkeys.

Parameter	C57BL/6 Mice 10 mg/kg			Cynomolgus Monkey 15 mg/kg		
	FGF21 Sandwich	GCG/FGF21	IL-1RA/FGF21	FGF21 Sandwich	GCG/FGF21	IL-1RA/FGF21
AUC_last_ (μg·hr/mL)	147.9	117.5	114.5	1789.2	883.4	778.4
C_max_ (μg/mL)	12.3	10.9	9.9	57.1	36.6	36.8
T_max_ (hr)	8	4	4	8	8	8
CL/F (ml/hr/kg)	67.6	85.1	8.7	8.3	17.0	19.3
t_1/2_,app (hr)	18.4	11.2	5.5	62.7	38.7	16.3

## Data Availability

Data are contained within the article or Supplementary Materials.

## Supplementary Materials

The following supporting information can be downloaded at: https://www.mdpi.com/article/10.3390/ph16111601/s1, Figure S1: Evaluation of various formats using an acceptor with a Fc carrier.
