# Supplementary material for: The Novel Tetra-Specific Drug C-192, Conjugated Using UniStac, Alleviates Non-Alcoholic Steatohepatitis in an MCD Diet-Induced Mouse Model
Source: Pharmaceuticals (Basel). 2023 Nov 13;16(11):1601. doi: 10.3390/ph16111601 (PMC10674394; doi:10.3390/ph16111601)
Supplement: Supplementary file 1 [file pharmaceuticals-16-01601-s001.zip › pharmaceuticals-2632718-supplementary.pdf]

Supplementary Materials

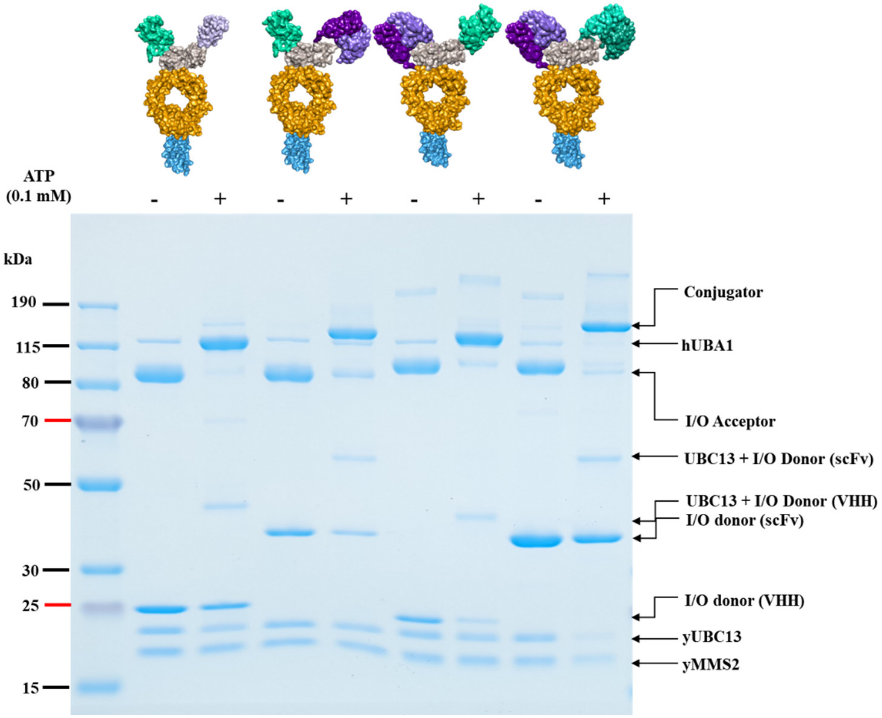

Figure S1. Evaluation of various formats using acceptor with Fc carrier. SDS-PAGE analysis showed that combination yield of acceptor and donor such as scFV, VHH, and cytokine through UniStac platform.
